# Comparative Genomics and Association Mapping Approaches for Blast Resistant Genes in Finger Millet Using SSRs

**DOI:** 10.1371/journal.pone.0099182

**Published:** 2014-06-10

**Authors:** B. Kalyana Babu, Pandey Dinesh, Pawan K. Agrawal, S. Sood, C. Chandrashekara, Jagadish C. Bhatt, Anil Kumar

**Affiliations:** 1 Department of Molecular Biology and Genetic Engineering, College of Basic Sciences & Humanities, G.B. Pant University of Agriculture and Technology, Pantnagar, Uttarakhand, India; 2 Biotechnology laboratory, Vivekananda Parvateeya Krishi Anusanthan Sansthan (VPKAS), Almora, Uttarakhand, India; National Institute of Plant Genome Research, India

## Abstract

The major limiting factor for production and productivity of finger millet crop is blast disease caused by *Magnaporthe grisea*. Since, the genome sequence information available in finger millet crop is scarce, comparative genomics plays a very important role in identification of genes/QTLs linked to the blast resistance genes using SSR markers. In the present study, a total of 58 genic SSRs were developed for use in genetic analysis of a global collection of 190 finger millet genotypes. The 58 SSRs yielded ninety five scorable alleles and the polymorphism information content varied from 0.186 to 0.677 at an average of 0.385. The gene diversity was in the range of 0.208 to 0.726 with an average of 0.487. Association mapping for blast resistance was done using 104 SSR markers which identified four QTLs for finger blast and one QTL for neck blast resistance. The genomic marker RM262 and genic marker FMBLEST32 were linked to finger blast disease at a P value of 0.007 and explained phenotypic variance (R^2^) of 10% and 8% respectively. The genomic marker UGEP81 was associated to finger blast at a P value of 0.009 and explained 7.5% of R^2^. The QTLs for neck blast was associated with the genomic SSR marker UGEP18 at a P value of 0.01, which explained 11% of R^2^. Three QTLs for blast resistance were found common by using both GLM and MLM approaches. The resistant alleles were found to be present mostly in the exotic genotypes. Among the genotypes of NW Himalayan region of India, VHC3997, VHC3996 and VHC3930 were found highly resistant, which may be effectively used as parents for developing blast resistant cultivars in the NW Himalayan region of India. The markers linked to the QTLs for blast resistance in the present study can be further used for cloning of the full length gene, fine mapping and their further use in the marker assisted breeding programmes for introgression of blast resistant alleles into locally adapted cultivars.

## Introduction

Finger millet (*Eleusine coracana* (L.) Gaertn.) (2n = 4 x = 36), sub-species *coracana*, belongs to the family Poaceae, genus *Eleusine* in the tribe Eragrostideae. Finger millet commonly known as *ragi* (India), *bulo* (Uganda), *wimbi* (Swahili), and *tellebun* (Sudan) is an important food crop cultivated widely in arid and semi-arid regions of the world, especially in East Africa, India and in other Asian countries including Sri Lanka and China [1]. It is believed that Uganda or a neighboring region is the centre of origin of *Eleusine coracana* and it was introduced to India, probably over 3000 years ago. The crop is grown mainly by subsistence farmers, which serves as a food security crop because of high nutritional value and excellent storage qualities [2]. One of the major limiting factors for production and productivity of finger millet crop is blast disease caused by *Magnaporthe grisea* (anamorph *Pyricularia grisea*). This disease has been identified as the highest priority constraint to finger millet production in Eastern Africa, and India since most of the genotypes are highly susceptible [3]. The causal organism of blast disease *Magnaporthe grisea* is also a causative agent of rice blast. The average loss due to blast disease has been reported to be around 28–36% [4], and in certain areas yield losses could be as high as 80–90% [5]. The disease affects the crop at all growth stages however, neck blast and finger blast are the most destructive forms of disease [6]. Disease resistance is frequently governed by specific recognition between pathogen avirulence (*Avr*) genes and corresponding plant disease resistance (*R*) genes. This type of gene for gene interaction usually is accompanied by a hypersensitive response leading to the restriction of pathogen growth [7]. Only nine R-genes (*Pita, Pi9, Pi2, Piz-t, Pi-kh, Pi36* and *Pi37*) have been isolated by different cloning strategies in rice, which confer resistance to blast belong to nucleotide binding site – leusine rich repeat (NBS-LRR) family. The NBS-LRR disease resistance genes belongs to a large and diverse super family of genes, which can be subdivided based on the characteristic N-terminal features of their products [8]. Conserved amino acid sequence motifs in the NBS domain have been widely used to isolate and classify NBS-LRR encoding genes. Growing cultivars with durable resistance is the best means of combating the blast disease of finger millet. The conventional breeding approaches are time consuming, dependent on environment and not accurate in the identification of genes for blast resistance which can be solved by molecular marker technologies.

With the advancement of molecular marker technology, a lot of progress has been made in majority of the crops except in the neglected crops like small millets, such as finger millet, though it is highly nutritious crop. A considerable amount of molecular work has been carried out since the establishment of molecular biology techniques [9], [10]. Dida et al. [11] generated the first ever genetic map of the finger millet genome using different types of markers like RFLP, AFLP, EST and SSR markers. The Expressed sequence tag (EST) projects have generated a vast amount of sequence data that are publicly available, which can be mined for simple sequence repeats (SSRs). The EST databases have become particularly attractive resources for such *in-silico* mining of SSRs, as was reported in cereal crops [12], [13], which can be further effectively used in diversity analysis, linkage map construction, QTL mapping studies [14] and marker assisted breeding programmes for disease resistance and quality improvement. However, there is very less amount of ESTs (1956) available for finger millet in comparison to other major cereals. Hence, comparative genomics plays very important role in such under-utilized crops like finger millet. The already available full sequence information of rice made it possible to identify the genes influencing the blast disease resistance in finger millet crop through comparative genomics.

Most traits of agricultural importance like blast disease are controlled by multiple quantitative trait loci (i.e. complex traits). Genetic mapping and molecular characterization of these functional loci facilitates genome-aided breeding for finger millet crop improvement. Two of the most commonly used tools for dissecting complex traits are linkage analysis and association mapping [15]. By exploring deeper population genealogy rather than family pedigree, association mapping offers few advantages over linkage analysis such as much higher mapping resolution, greater allele number, broader reference population, and less research time in establishing an association [16]. In finger millet till now there are no reports on the molecular characterization and mapping of blast resistant genes. So, there is a need to identify the molecular markers linked to the blast resistance for their further introgression into locally well adapted germplasm. With this aim, the present study was conducted on a global collection of 190 finger millet accessions belonging to different parts of the world including ICRISAT mini-core collection for 1) Comparative genomic analysis for blast resistance genes of finger millet with rice, 2) Phenotyping of finger miller genotypes for leaf, neck and finger blast disease, 3) Population structure analysis of finger millet genotypes for blast resistance using genomic and genic SSRs, and 4) Association mapping of blast resistant genes in finger millet genotypes using genic and genomic SSRs.

## Materials and Methods

### Plant materials and experiments

In the present study, a total of 190 finger millet accessions belonging to different regions of the world *viz.,* African continent (Zimbabwe, Kenya, Maldives, Uganda, Malawi, Senegal, Nigeria, and Zambia), South Asian continent (India and Nepal) and Germany were used. The finger millet germplasm was collected from different sources, which included 84 accessions from the ICRISAT mini-core gene bank, ICRISAT, Hyderabad, 64 accessions from Gobind Ballabh Pant University of Agriculture and Technology (G.B.P.U.A&T), Pantnagar, while 42 accessions from Vivekanada Parvatiya Krishi Anusanthan Sansthan (VPKAS), Almora (Table S1). The finger millet accessions were grown under mid-hill condition at VPKAS experimental farm, Hawalbagh, Almora (29^0^ 36′N and 79^0^ 30′N situated at an elevation of 1,250 m above mean sea level) in augmented block design with two checks VR708 and RAU8 replicated in 10 blocks (19 entries in each block and the checks were replicated in each block). Recommended agronomic practices (N: P: K 40∶20∶0) were followed for raising the crops.

### Field screening for blast disease severity assessment

The 190 finger millet accessions along with checks (VR 708, and RAU 8) were evaluated in the finger millet blast nursery at VPKAS, Almora, Uttarakhand, India. The blast disease severity assessment was done as per the earlier reports [4].

### DNA extraction and quantification

The genomic DNA of 190 finger millet genotypes was isolated using standard protocol [17]. After extraction, 1 µl of DNA sample of all accessions was loaded in 0.8% agarose gels. Uncut lamda DNA was loaded as a control to assess the quality and the quantity of DNA. Based on uncut lamda DNA standards, DNA samples were normalized to a uniform concentration (25 ng/µl) for SSR genotyping.

### SSR amplification and detection

The polymerase chain reactions (PCR) were performed in 20 µL reaction volume containing 2 µL of 10× buffer having 15 mM MgCl2, 0.2 µM of each forward and reverse primer, 2 µL of 2 mM dNTPs, 0.2 µL of 1 U of *Taq* DNA polymerase (Invitrogen, USA), and about 25 - 50 ng of template DNA. The PCR amplification protocol was standardized for genomic SSRs and blast specific functional markers. The genomic SSRs used in the present study were obtained from earlier studies [2]. For genomic SSRs, the amplifications were performed in a Thermo cycler (MJ Research, USA) programmed for an initial denaturation of 3 min at 95°C followed by 35 cycles of 30 s at 95°C, 30 s of annealing temperature (different annealing temperatures for different primers), extension of 1.0 min at 72°C, with a final extension of 10 min at 72°C, and hold at 4°C. The PCR reactions for blast specific genic markers were programmed for an initial denaturation of 5 min at 94°C followed by 40 cycles of 1 min at 94°C, 1.5 min at different annealing temperatures for different primers, 2.0 min at 72°C, with a final extension of 7 min at 72°C, and hold at 4°C. The PCR products were fractioned on 3.5% Super Fine Resolution (SFR) agarose gel. The electrophoresis was held at 100 volts for 3 h at room temperature. Gels were stained with ethidium bromide and visualized using Bio Imaging System (SynGene, USA). Only those bands that were clear and reproducible were scored for data analysis. The repeatability of the scored bands was ensured by repeating the PCR and scoring of bands by two individuals. Molecular weight of the bands was estimated using 100 bp DNA ladder as standard.

### Expressed sequence data (EST) based SSR mining for blast genes

The EST sequences for blast related genes like NBS-LRR regions from finger millet were down loaded in FASTA format from NCBI website. A total of 57 EST sequences were downloaded and used for primer designing. These sequences were further used for BLASTn analysis to find the homologs in the rice database. Also sequences of various rice blast resistance genes *i.e.*, *Pi* genes were also downloaded. The downloaded sequences were obtained in FASTA format for sequence assembly and SSR analysis. The SSR identification was done using one pipeline tool “Websat” software [18]. Primers designed to flanking sequences using the “Websat” software which uses the Primer3 software. Six classes of SSRs, *i.e.,* mono-, di-, tri-, tetra-, penta-, and hexa nucleotide repeats were targeted for identification using this tool. The type of search criteria taken for identification of EST-SSRs was minimum number of repeats as 10 for mononucleotide, 6 for di nucleotide, 4 for tri nucleotide, and three for tetra, penta and hexa nucleotides. The main parameters for primer designing were, GC content of 40–60%, annealing temperature (*Tm*) of 50–60°C and expected amplified products size of 100–450 bp. All other parameters were set to default values.

### Strategy of comparative analysis of NBS-LRR regions

The EST sequences of finger millet were retrieved from the NCBI website. These sequences were used for BLASTn analysis to identify the homologous *Oryza sativa* sequences at an E value more than 4-e13. The rice *Pi* gene sequences were also downloaded for the primer designing. The positions of these EST sequences was identified on the rice chromosome maps (www.gramene.org) and compared with the positions of different rice blast genes. The tentative position of the markers on the finger millet chromosomes were derived as per the comparative mapping studies of Srinivasachary et al. [19].

### Nucleotide sequencing by ABI 3130XL genetic analyzer

All primer pairs were initially tested via PCR and agarose gel analysis for identifying those pairs producing single amplicons. The primers producing single amplicons, showed polymorphism between resistant and susceptible genotypes for blast disease resistance was used for further analysis. The PCR products were purified using a QIAquick PCR purification kit (Qiagen Inc., Valencia, CA, USA) according to the manufacturer's protocol. Dideoxy cycle sequencing was performed using the chain-termination method and an ABI Prism Big Dye reaction kit (ver. 3.1) according to the manufacturer's protocols (Applied Biosystems). The sequencing products were run on an ABI 3130XL Genetic Analyzer. Sequence editing and assembly of the contigs were performed using Sequencher 4.10. For comparisons among the sequences of genotypes, BioEdit (ver. 7.0.5.3) [20] with the ClustalW multiple alignment option was used and adjusted manually by the authors. The PCR products were sequenced from both ends and the resulting termination products were analyzed on an ABI 3130XL Genetic Analyzer. The two resulting sequence traces derived from opposite ends of each amplicon were analyzed, aligned with standard DNA analysis software Phred and Phrap (http://www.phrap.org/).

### Phylogenetic analysis

Phylogenetic analyses of the nucleotide sequences were performed by multiple alignments of nucleotide sequences using the ClustalW program [21]. The phylograms were drawn with the MEGA4 program using either NJ (Neighbor Joining) or UPGMA (Unweighted Pair-Group Method with Arithmetic Mean) methods [22].

### Data analysis

The data set of SSRs on 190 accessions were used for statistical analysis using Power Marker V3.0 software [23] for estimating basic statistics *viz.,* PIC value, allelic richness as determined by the total number of the detected alleles and a number of alleles per locus, gene diversity (*He*), occurrence of unique, rare, common alleles, and multiple allele percentage. The data were tested for presence of population structure and analysis of molecular variance (AMOVA) to separate the total molecular variance into components between groups, within groups and the significance of F_ST_ using GenAlEx 6.5 software [24].

### Population structure analysis

Analysis of the population structure and gene flow between finger millet accessions was carried out using a model based clustering method as implemented in the software programme STRUCTURE v. 2.3.4 [25]. In this method, it is assumed that number of subpopulations exists in the sample analyzed. Each accession can have membership in different subgroups (admixture model; ALPHAPROPSD = 0.20). The number of subgroups (K) in the population was determined by running the programme at different K values with K varying from 1 to 10 with five independent runs for each K value. We used a burn-in period of 1,00,000 and 10,00,000 replications. The clustering pattern of genotypes was also done using the principal component analysis (PCA) software of NTSYS v2.02 [26].

### Association of markers with phenotypic data

Association analysis was done by using phenotypic data of global collection of finger millet genotypes, genotypic data of 104 SSR marker data and population structure data (Q matrix) by using software TASSEL [27]. The marker–trait association analysis was conducted using TASSEL 3.0 software along with the general linear model (GLM) and mixed linear model (MLM) procedures. The kinship matrix was used in addition to the genotypic, phenotypic and Q matrix data in the MLM approach. The significant threshold for the association was set at P<0.01 and <0.001.

## Results and Discussion

### Comparative genomic analysis for blast resistance genes of finger millet with rice

Finger millet is a highly neglected, under-utilized crop and the information on EST sequences available for finger millet is scarce. Hence, there is a need to explore the comparative genomic strategy to identify the molecular markers associated with important agronomic traits like blast resistance of finger millet by comparing with fully sequenced genome like rice. With this aim, a total of 58 SSRs were designed from 82 GenBank accessions representing the different genes influencing the blast resistance in rice and finger millet from CDS, 5′UTR, 3′UTR and intron regions of the sequences. These EST sequences belonged to NBS-LRR region (for blast resistance) of rice and finger millet, rice *M. griseae* genes, and rice blast genes such as *Pi-ta, Piz, Pi1, Pi2, Pi3, Pi4, Pi5, Pi14, Pi16, Pi21, Pi25*. The details of the designed EST-SSRs used in the study along with their source, repeat motif, expected product size, and homologous gene function has been given in table S2. Five genic SSR markers were designed from the finger millet NBS-LRR region, whereas 12 primer pairs were designed from the rice NBS-LRR region which includes the blast resistance gene sequences also. Seven primer combinations were designed from the *M. griseae* genes of rice, while 14 primer pairs were developed from several cloned genes of rice blast genes (*Pi-ta, Piz, Pi1-5, Pi21, Pi25, Pi14* and *Pi16*). The hypothetical chromosomal location of the primers designed from the EST sequences and the rice SSR loci was given in figure S1. All the primers were spread among the nine out of 12 chromosomes of rice, however they spread across eight out of nine homeologous chromosomes of finger millet. Most of the primers were located on the rice chromosomes 2, 6, 9, 11 and 12, whereas they were located on chromosomes 2, 4 and 6 of finger millet genome (Figure S1).

**Figure 1:**
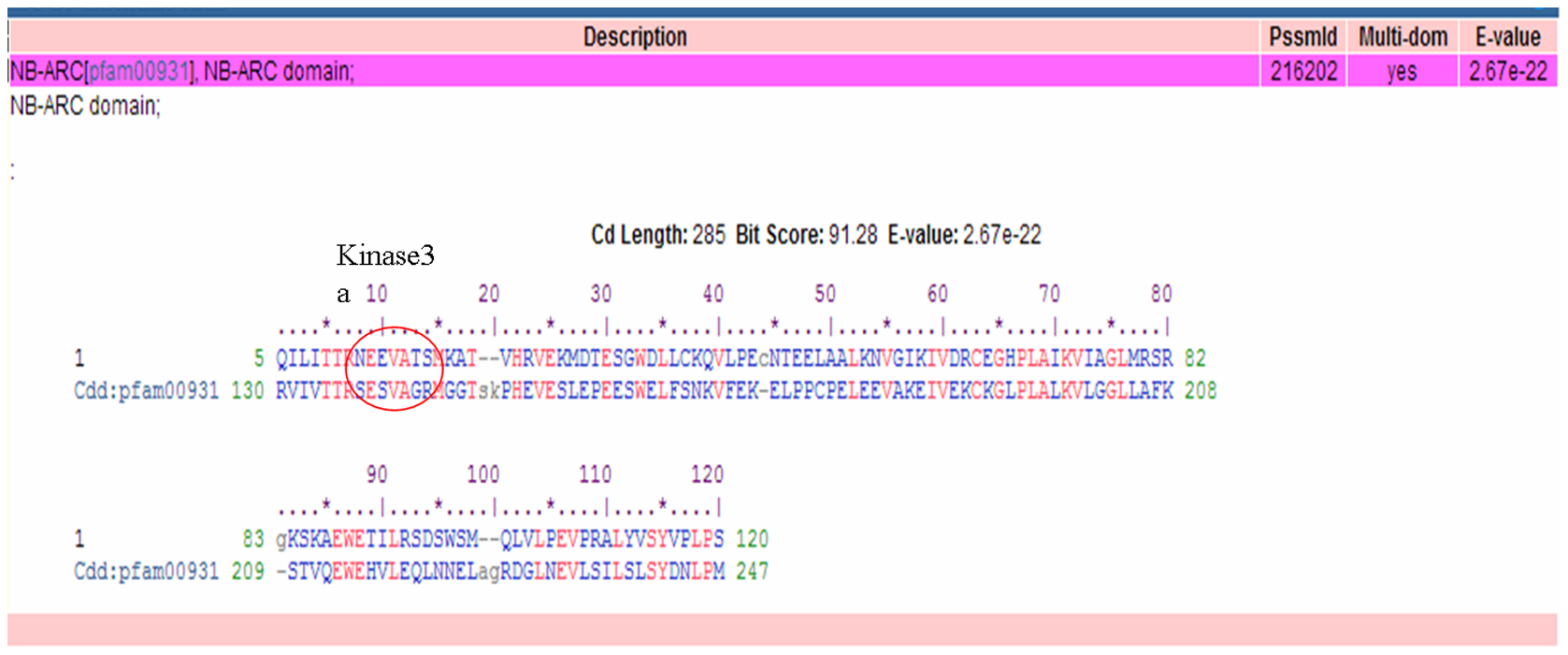
The kinase3a motif of NB–ARC domain present in the sequence of finger millet genotype as obtained from CD domain search.

The sequences obtained from resistant and susceptible genotypes were performed BLASTn analysis to find the homologous sequences (submitted to NCBI). The sequences from resistant genotypes found high similarity with the rice blast *Pi* genes and NBS-LRR regions while the sequence obtained from the susceptible genotypes did not show any similarity with the any NBS-LRR region (Table 1). The sequences of the resistant alleles were located on 2^nd^ and 11^th^ chromosomes of rice. This was supported by the earlier reports in rice, where most of the blast resistant genes have been mapped on 2^nd^, 6^th^, 11^th^ and 12^th^ chromosomes of rice [28]. Our results also showed that 2^nd^ and 11^th^ chromosomal region sequences of rice could be playing important role in the blast disease resistance of finger millet. The nucleotide sequences were further analyzed by conserved domain (CD) search available in the NCBI website which resulted in identification of the NB-ARC (nucleotide binding-APAF-1, R proteins, and CED-4) domain in our sequences. The amino acid sequence of finger millet resistant genotype was further compared with the previously cloned plant disease resistance genes which showed the characteristic NBS motifs of kinase-2 and kinase 3a of plant R-genes (Figure 1), confirming that the sequences characterized in the present study belonged to the NBS-LRR gene super family.

**Table 1:** The details of the sequences obtained from sequencing along with chromosomal location, conserved domains.

Accession	Sequence ID	Primer	Length (bp)	Homologous sequences	Conserved domains	S/R	Chromosomal location
GE2136	GBFMB1	FMBLEST2	401	EU075256-263, EU075253, EF408629.1	NB-ARC domain	R	2
GE554	GBFMB2	FMBLEST2	679	EU075256-263, EU075253, EF408629.1	NB-ARC domain	R	2
GE1146	GBFMB3	FMBLEST2	530	EU075256-263, EU075253, EF408629.1	NB-ARC domain	R	2
VR708	GBFMB4	FMBLEST2	563	No similarity found with resistant sequences, since it is Susceptible genotype	NO	S	4
VL315	GBFMB5	FMBLEST5	654	Y09810, Y09809, AF032692.1, EU293163.1, AY518220, AY337867.1	NB-ARC domain	R	11
VR708	GBFMB6	FMBLEST5	432	AC121352, AC111015.2, ZM001062669.2	NO	MR	5
VL333	GBFMB7	FMBLEST5	554	DQ272576.1, Y09812.1, AF59886.1, AY169509.1, AY518220.1, AF159885.1 EU075252.1, EU075247.1, EU075246.1, EU075238.1, EU075239.1, EU075223.1-226	NB-ARC domain	R	

### Phenotyping of finger millet genotypes for blast resistance and their correlation

A global collection of 190 finger millet genotypes were evaluated for three types of blast resistance *viz.*, leaf blast, neck blast and finger blast under natural field conditions. The field which was used for blast screening was known to be a hot spot of severe occurrence of blast disease on both finger millet and rice. Hence, under natural conditions the finger millet genotypes were screened for blast disease resistance.

### Neck blast resistance

The finger millet genotypes were grouped in to four groups (HR, R, MR-MS, and S-HS) based on the disease score for neck blast. Based on the mean neck blast severity, 94 accessions were found highly resistant (HR)/resistant (R), 78 were resistant to moderately resistant (MR), 15 genotypes were susceptible (S) and three genotypes were highly susceptible (HS) compared to the susceptible check VR708 and resistant check RAU 8. The genotypes VHC3917, VHC3939 and GPHCPB26 were highly susceptible to neck blast and all three were belonged to NW Himalayan region of India. Most of the exotic genotypes including ICRISAT mini core collection were highly resistant to moderately resistant, except the genotype from Nepal (IE6082) which was susceptible to neck blast. The susceptibility of the IE6082 genotype to neck blast may be due to the proximity of Nepal to NW Himalayan region and there might be germplasm exchange between Nepal and the NW Himalayan region of India. Similar conclusions were drawn from the recent studies [29], where they also reported that 68 (out of 80) accessions of the ICRISAT mini-core collections were found resistant and six were moderately resistant to the neck blast. The genotypes susceptible to neck blast were IE501, GE1621, GE1583, VHC 3903, VHC3893, VL315, VHC3970, VHC3951, VHC3697, GPHCPB5, GPHCPB20, and GPHCPB27. Most of genotypes were from NW Himalayan region of India. Among the genotypes of NW Himalayan region of India, VHC3997 and VHC3930 were found highly resistant to neck blast.

Neck blast found to have significant and positive correlation with finger blast (0.612**), but a poor correlation were observed with leaf blast (0.08**). Our results were similar to the earlier studies [30], where they also found poor correlation between leaf blast and neck blast under natural conditions. The high and significant correlation (0.920**) between neck blast and finger blast has also been reported in some recent studies under artificial inoculations [29]. Moderate correlation of leaf blast with neck and finger blast suggested that leaf blast severity in the early stages may not result in severe neck or finger blast during the later stages of plant development. It has been reported that seedlings of finger millet were more susceptible to leaf blast than mature plant [31]. However no relationship was known between the intensity of seedling infection and that of neck and finger infection. Contrasting responses between the vegetative stage and reproductive stage often occur, indicating differential gene expression for resistance to leaf, neck and finger blast [32].

### Finger blast resistance

The finger millet genotypes were grouped in to four groups (HR, R, MR-MS, and S-HS) based on the disease score for finger blast. Based on mean finger blast severity, a total of 149 finger millet genotypes were found resistant to highly resistant, 32 were moderately resistant, while only 9 were found susceptible compared to the susceptible check VR708 and resistant check RAU 8. The genotypes IE501, VHC3917, VHC3939, GPHCPB1, VHC3870, VHC3970, GPHCPB13, GPHCPB20, and GPHCPB26 were found highly susceptible to finger blast disease. All these genotypes belonged to NW Himalayan region of India. The susceptibility reaction of these genotypes may be due to earliness in maturity, since the conditions favoring disease matches the reproductive phase of these genotypes. Therefore, most of the medium and late duration genotypes escape the disease even if they are susceptible. Among the genotypes from NW Himalayan region of India, the accessions, VHC3997, VL324, and VHC3996 were highly resistant to finger blast. However, the exotic genotypes were found to be resistant to the finger blast like neck blast disease which was well supported by earlier reports [29].

### Leaf blast resistance

Based on mean leaf blast severity, only 13 genotypes (IE4121, IE2872, IE5066, IE2043, IE3317, IE2871, IE2217, IE3470, IE6154, IE4646, IE3470, GPHCPB11 and IE2589) found to show resistant reaction, while the remaining all 177 genotypes showed susceptible to moderately resistant/susceptible reaction. The genotypes resistant to leaf blast also showed resistant reaction to finger blast and neck blast. Under favorable conditions, leaf/foliar blast occurred in majority of accessions at the seedling stage, which did not correlate well with crop growth stages and maturity of plants, probably because of the build-up of adult plant resistance. Hence, neck and finger blast reaction were considered important parameters for blast resistance [4], [31]. It was positively and significantly correlated with finger blast (0.227**).

### Genetic analysis of finger millet genotypes for blast resistance using functional SSRs

A total of 58 SSR markers, of which 43 genic SSR markers designed from the EST sequences of different blast genes and 15 rice genomic SSRs tightly linked to blast QTLs [32] have been used in the present study to investigate the genetic variation among the 190 finger millet genotypes for blast disease severity. The genomic DNA of 190 finger millet genotypes was amplified using 58 SSR markers. These 58 SSRs were spread across the chromosomes of finger millet genome. The 58 SSRs yielded ninety five scorable alleles, of which sixty-five were found polymorphic. A total of 28 (49%), out of the 58 SSRs were found polymorphic and the remaining 30 (51%) were monomorphic. This low level of polymorphism may be due to self pollinated nature of the finger millet crop and also the polymorphism ability of the genic SSR markers was less compared to the genomic SSRs. As expected, the amount of marker polymorphism (49%) exhibited in the finger millet composite collection was higher than normally found in self pollinated cereal crop species like rice [33] and Wheat [34]. Normally in inbreeding species, the level of polymorphism is expected to be lower than out crossing species [35].

The 28 polymorphic markers yielded 65 scorable alleles with a mean of 2.4 alleles per marker, whereas it was 1.7 alleles per marker including the monomorphic SSRs used in the study. The number of alleles generated with the polymorphic loci ranged from two to a maximum of four among the finger millet genotypes of the study. Two SSRs *viz.*, RM5963 and RM23842 were found to have maximum number of alleles (4 each), while five (FMBLEST8, FMBLEST32, FMBLEST33, RM262, and RM23808) had three alleles each (Table 2). All the remaining SSRs (21) had only two alleles. Likewise, some workers analyzed a set of 11 elite finger millet genotypes using 31 SSRs designed from the EST sequences available at NCBI and found only 17 were polymorphic [36]. Similarly, Panwar et al. [37] also found 50% of the markers were polymorphic, however, they got 6.8 alleles per primer. Reddy et al. [38] designed 30 EST based SSR loci from the EST sequences available at NCBI and found 20 were polymorphic. Recently, Bharathi, [39] studied a large set of finger millet genotypes for their agro-morphological traits and found 20 were polymorphic out of the 30 genomic SSRs, which generated alleles from the range of 7 to 25 at an average of 11.55 alleles per locus. This high number of alleles may be due to a very large collection of genotypes used in their study (more than 900). These results indicated that genic microsatellite loci were less efficient than genomic SSR loci for polymorphism analysis. However, since these were gene specific SSR loci, the polymorphism detected maximum belongs to the gene of interest. A conclusion may be derived that the loci with more number of alleles can be very useful in the assessment of genetic diversity.

**Table 2:** The polymorphism details of major allele frequency, allele number, gene diversity, heterozygosity and PIC values of the blast specific genic SSRs (naming of FMBLEST- finger millet blast EST).

Marker	Major allele frequency	Allele Number	Gene Diversity	Heterozygosity	PIC	Inbreeding coefficient (f)
FMBLEST2	0.581	2.000	0.487	0.000	0.368	1.000
FMBLEST4	0.538	3.000	0.497	0.000	0.374	1.000
FMBLEST5	0.882	2.000	0.208	0.221	0.186	−0.059
FMBLEST8	0.512	3.000	0.508	0.520	0.387	−0.021
FMBLEST10	0.503	2.000	0.500	0.000	0.375	1.000
FMBLEST12	0.521	2.000	0.499	0.000	0.375	1.000
FMBLEST15	0.620	2.000	0.471	0.000	0.360	1.000
FMBLEST17	0.613	2.000	0.475	0.763	0.362	−0.606
FMBLEST19	0.536	2.000	0.497	0.027	0.374	0.947
FMBLEST32	0.600	3.000	0.519	0.112	0.430	0.786
FMBLEST33	0.616	3.000	0.476	0.007	0.366	0.985
FMBLEST34	0.690	2.000	0.428	0.000	0.336	1.000
FMBLEST35	0.602	2.000	0.479	0.057	0.364	0.881
FMBLEST36	0.628	2.000	0.467	0.619	0.358	−0.323
FMBLEST40	0.567	2.000	0.491	0.000	0.370	1.000
FMBLEST41	0.561	2.000	0.493	0.816	0.371	−0.655
FMBLEST42	0.571	2.000	0.490	0.000	0.370	1.000
FMBLEST43	0.576	2.000	0.488	0.000	0.369	1.000
RM262	0.425	3.000	0.623	0.824	0.543	−0.318
RM3330	0.688	2.000	0.429	0.582	0.337	−0.354
RM5963	0.375	4.000	0.726	0.750	0.677	−0.023
RM3148	0.582	2.000	0.487	0.000	0.368	1.000
RM10076	0.770	2.000	0.355	0.383	0.292	−0.077
RM17827	0.635	2.000	0.464	0.000	0.356	1.000
RM23808	0.503	3.000	0.546	0.048	0.443	0.912
RM23842	0.443	4.000	0.651	0.885	0.579	−0.358
RM21	0.703	2.000	0.418	0.311	0.331	0.259
RM254	0.646	2.000	0.457	0.708	0.353	−0.542
Mean	0.589	2.321	0.487	0.273	0.385	0.443
MIN	0.375	2.000	0.208	0.000	0.186	−0.655
MAX	0.882	4.000	0.726	0.885	0.677	1.000

The polymorphism information content (PIC) demonstrates the informativeness of the SSR markers and their potential to detect differences among the genotypes based on their genetic relationships. The PIC values of all the polymorphic markers across the 190 finger millet genotypes varied from 0.186 to 0.677 at an average of 0.385 (Table 2). Similar results have been obtained by some workers, where they reported a slightly higher PIC in the range of 0.274 to 0.758 for the analysis of variation for calcium content among a set of 52 finger millet genotypes [37]. Bharathi [39] also used genomic SSRs for the diversity analysis and found PIC in the range of 0.196 to 0.834. This showed that the results obtained in our study were largely in congruence with the earlier studies [37], [39]. But, some workers found higher PIC values than our results by using the EST based SSR primers which could be attributed to more repeats of the repeat motifs observed in the EST-SSR primers [38]. The maximum PIC value was observed for SSR marker RM5963 (0.677) followed by RM23842 (0.579) and RM262 (0.543). The lowest PIC value was observed for the SSR marker FMBLEST5 (0.186) followed by RM10076 (0.292). The SSRs namely, RM5963, RM23842, RM262 and FMEST32 are noteworthy due to their relatively higher level of polymorphism. These four SSR markers can be effectively used in further finger millet crop improvement breeding programmes for the enhancement of blast disease resistance. We showed that the genomic SSR markers tightly linked to the blast resistance were able to generate more polymorphism than the functional SSR markers. Five SSR markers had the PIC range of 0.400 to 0.677 with an average value of 0.540, while 23 SSRs had the PIC range of 0.00 to 0.40 with an average of 0.353. A close proportionate relationship between the number of alleles and the PIC values of SSRs was observed in the present investigation. For example the SSRs RM5963 and RM23842 were found to have high PIC values along with more number of alleles (4).

### Statistical analysis of genetic diversity using SSR markers

Gene diversity also known as ‘expected heterozygosity’ (*He*) is defined as the probability that two randomly chosen alleles from the population are different. Functional markers such as EST-SSR assay polymorphism associated with the coding regions of the genome can detect “true gene diversity” available inside or adjacent to the genes [40]. In the present study, the gene diversity was in the range of 0.208 to 0.726 with an average value of 0.487 (Table 2). Similar results were also obtained in earlier studies, where they found the variation of *He* value from 0.200 to 0.850 [39]. However, there are reports of very less gene diversity (0.024 to 0.327) also, which may be due to less number of markers used in the study [41]. The expected heterozygosity was found to be highest with the SSRs RM5963 (0.726), followed by RM23842 (0.651), and RM262 (0.623). The gene diversity present among the finger millet genotypes showed that markers used in the present study were more polymorphic. Similar conclusions were obtained in other cereal crops particularly maize where they found gene diversity in the range of 0.11 to 0.81 with an average of 0.59 [42]. The lowest gene diversity was found in the SSRs FMBLEST5 (0.208) followed by RM10076 (0.355). A total of 16 SSRs were observed to have more gene diversity than the average value 0.487 while, 12 SSRs had the *He* less than the average value. The SSRs which had more alleles and high PIC, also showed higher gene diversity. These results showed that PIC values are proportionately correlated with the number of alleles and the gene diversity [43].

The heterozygosity, known as ‘observed heterozygosity’ (*Ho*) was observed at an average of 0.221 and ranged from 0.00 to 0.885 which showed a wide range of heterozygosity was present in the finger millet genotypes, which was similar to the earlier reports in maize [42]. The *Ho* was highest in the SSR marker RM23842 (0.885), followed by RM262 (0.824), and FMBLEST41 (0.816). The reason for such high heterozygosity would be allotetraploid nature of the finger millet having A and B genomes. The locus UGEP3 was mapped in both the genomes and this could be the reason for occurrence of two alleles and high heterozygosity for this locus [2]. Also the involvement of large number of Indo-African lines in the study may be the possible reason for high heterozygosity due to residual heterozygosity at least in some of the SSR loci. The heterozygosity observed at some of the loci could also be due to high mutation rate and mutational bias at SSR loci [44]. The SSRs with large number of repeat units tend to show high mutational rate. As a result, any mutations in any one of the alleles may create a heterozygous condition [45].

### Population genetic estimates by AMOVA analysis

For the purpose of AMOVA and F_ST_ estimates, the finger millet genotypes were grouped according to their blast disease response. AMOVA has clearly brought out significant differences among various genotypes evaluated. It was observed that greater variance (72%) was observed by individuals within population, while between the populations it was less (28%) (Table 3). The pair wise fixation indices (F_ST_) among the groups were given below the diagonal of the table 4. The highest pair wise F_ST_ was observed between population 1 and 3 (0.328), while the lowest was recorded between P2 and P3 (0.092). Estimates of the fixation indices revealed a strong genetic structure between the populations 1 and 3, indicating the presence of strong population structure. The presence of strong genetic structure indicated that these two groups were reproductively and genetically isolated from each other. These two populations also showed greater variation for blast disease response. The population 1 was consisted of highly susceptible genotypes, whereas the population 3 consisted of resistant to HR genotypes.

**Table 3:** Analysis of molecular variation (AMOVA) of 190 finger millet genotypes based on 58 genic SSRs.

Source of variation	d.f.	Sum of squares	Variance of components	Percentage of variation
Among Populations	3	1225.400	8.443	28%
Within Populations	186	4131.048	22.210	72%
Total	189	5356.447	30.653	100%

**Table 4:** Pair wise F_ST_ estimates* among the four groups of finger millet genotypes.

Populations	pop1 (HS-MR)	pop2 (MS-MR)	pop3 (MR-R)	pop4 (HS-MR)
pop1	0.000	0.010	0.010	0.010
pop2	0.313	0.000	0.010	0.010
pop3	0.328	0.092	0.000	0.010
pop4	0.319	0.197	0.223	0.000

*Fst Values below diagonal. Probability values are shown above diagonal.

### Population structure analysis

**Figure 2:**
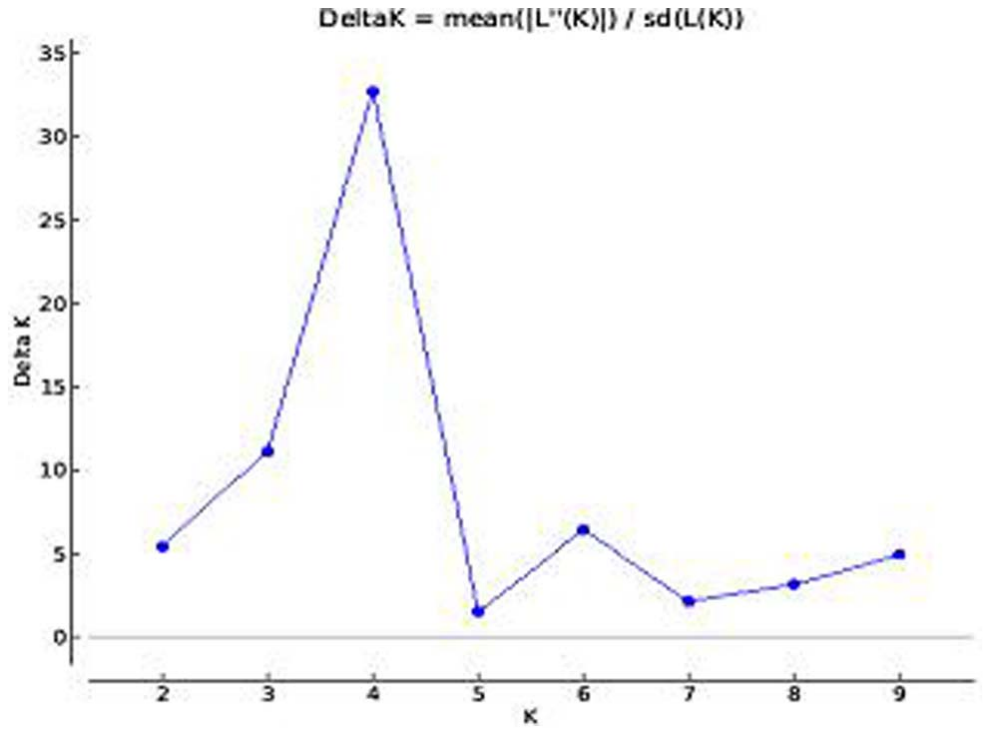
Identification of the appropriate sub-population number (K): Sub population number (K) against delta K and the maximum K value observed at K = 2.

The global finger millet collection representing different countries genotypes belonged to different countries (including India) were evaluated for estimation of population structure for finger millet blast disease resistance using a panel of 104 SSR markers spread across all the chromosomes. From the phylogenetic analysis, we came to know that there are at least four population groups, largely corresponding to their blast disease response (data not shown). The phylogenetic analysis has also provided some evidence for gene flow between these genotypes. For estimation of the exact population structure (*K*), Ks from 1 to 10 (with ten iterations) were ran and the LnP(D) value was used to group all the genotypes. The maximum ΔK value was observed for K = 4 (Figure 2). The number of groups was also validated using the principal component analysis. The PCA also resulted in grouping the finger millet genotypes into three clusters (HS-MR, MR-R and R-HR). In structure software, HS-MR genotypes were clustered into two groups, however in PCA analysis they were under single group. The inferred ancestry at K = 4 suggested that the finger millet genotypes were grouped into four populations. There was good correspondence between the phylogenetic tree, PCA analysis and the population structure in differentiating the finger millet genotypes into different clusters (HS-MR, MR-R and R-HR) based on their response to the blast disease. Although the population groups corresponded largely to their response to blast disease, there were some notable exceptions. The results of structure showed that all the four groups had admixture of alleles and no pure lines were observed. Hence, we have taken 20% criteria for considering as admixture in those genotypes.

**Figure 3:**
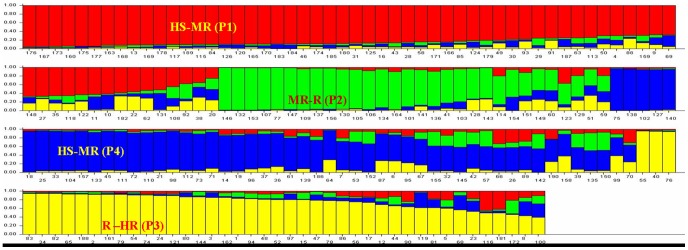
The population structure of 190 finger millet genotypes using the blast specific SSR loci revealed by STRUCTURE software (HS-Highly susceptible; MR-Moderately resistant; R- Resistant; HR- Highly resistant, for labels please refer to table S1).

The finger millet genotypes in population 1 (P1) and population 4 (P4) showed HS to MR type of response to the blast disease. The population 2 (P2) consisted of MR-R genotypes, whereas the population 3 consisted of R to HR genotypes (Figure 3). Till now, there are no reports on population structure analysis for blast disease resistance in finger millet. However, recently population structure was analyzed among 79 finger millet genotypes and showed that *E. coracana* germplasm formed three largely distinct sub populations, representing subsp. *africana, coracana* originating from Africa and *coracana* originated from Asia [11]. Their results were mostly according the geographic origin since they used the genome wide SSR markers. However, in our study we used the gene specific and genomic SSRs for blast resistance, hence grouped the finger millet genotypes based on the blast resistance. The HS-MR genotypes were grouped into two different populations (P1 and P4) which were similar to the power marker analysis. This further grouping of the HS-MR genotypes may be based on their geographic origin. The population 1 were mostly from different parts of India, where as population 4 comprised of genotypes mostly from NW Himalayan region of India (Figure 3). This showed the effectiveness of genic markers in differentiating finger millet genotypes solely based on their blast disease score. In the P1 population, twenty one genotypes found to have admixture of resistant and medium resistant alleles which was more than 20%. This may be due to involvement of exotic germplasm in the breeding programme of finger millet. During, 1970s, there was a lot of germplasm exchange between India and African countries for generating better genotypes. For example, the genotype IE1012 was an African cultivar, exploited in India as a source of blast resistance [46]. Out of the 21 genotypes, three genotypes IE4816, IE2430 and IE3794 were found to have more than 50% admixture of resistant alleles, and also showed the resistant reaction to the blast disease. Likewise the genotypes IE6337 and IE6240 (both from Zimbabwe) were resistant, moderately resistant respectively and contained 21% admixture of resistant alleles. This showed that these genotypes were resistant phenotypically as well as at molecular level though grouped with susceptible genotypes. This high percent of admixture was also found in earlier studies where they found 56% African and 43% Asian alleles in the genotype IE2980, a line from Sri Lanka [11]. Hence, it may be assumed that these alleles were sufficient to give the resistant reaction to the blast disease. Under the P1, only single genotype GPHCPB1 found to be pure line which was susceptible to the blast disease. It comprised of 99% alleles from the P1, and 1% alleles were from the P4 *i.e.,* HS-MR genotypes. So, this genotype can be considered as highly pure line which consisted of only the susceptible alleles. Such type of genotypes can be used in generating the mapping populations for fine mapping of blast genes. The genotype GPHCPB2 also had 99% alleles of HS germplasm, but it contained very minute number of resistant alleles (1%) which can be considered as nearly pure line and was found to be MR to the blast disease.

The population 2 (P2) comprising of 48 genotypes contained of mostly the MR-R genotypes. Under this group, percentage of admixture was less in comparison to other three groups. There were only four genotypes (VHC3895, VHC3865, GE3147 and IE4057), which contained more than 20% admixture of susceptible alleles. The genotype GE3147 (from India) was phenotypically found resistant, but had 45% susceptible alleles. This type of disease response is called as apparent resistance. Though it consisted of nearly equal amount of susceptible and resistant alleles, but phenotypically resistant reaction was observed to blast disease. The genotype VHC3865 consisted of 23% susceptible alleles, appeared to be moderately resistant. The genotype IE5106 from Zimbabwe had alleles from all the four populations equally (25%) which observed resistant reaction to finger millet blast disease. Three genotypes (VHC3930, VHC3873 and VHC3944) were found to be pure lines, representing to the NW Himalayan region of India.

The population 3 (P3) consisted of mostly resistant and highly resistant genotypes with few exceptions (GE116, GE356, GE384, IE3104, GPHCPB13). Fourteen genotypes were found to have admixture of population. Out of them, the genotypes GE116, GPHCPB13, GE1298, IE2710, IE3392 and GE356 contained considerable amount of admixture of susceptible and moderately susceptible alleles from other populations. Though the genotype GPHCPB13 was grouped with resistant genotypes, but phenotypically it showed susceptible reaction to blast, since it consisted of 50% susceptible alleles. This type of higher admixture between populations was also supported by earlier studies, where they found evidence of admixture of Asian accessions with African germplasm. Though the genotypes IE2710 and GE1298 had 48% susceptible alleles, they showed resistant reaction to blast disease phenotypically. This may be due to apparent resistance or the dominant effect of resistant alleles over the susceptible alleles. Remaining genotypes also had some level of admixture but it was negligible. The genotypes IE3618 and IE4734 can be considered as pure lines which had nearly 99.9% alleles from the same population which showed resistant response to the finger millet blast disease. The population 4 (P4) had a good amount of admixture of alleles from the other three populations. The P4 consisted of mostly HS-MR genotypes with few exceptions. Twenty two genotypes were found to have the admixture of alleles, of which IE5870, IE3797, IE4121, IE3795, and IE6294 genotypes were found to contain considerable amount of resistant alleles (more than 20%) and showed resistant reaction to blast disease.

The phylogenetic and structure analysis differed in few instances. In phylogenetic analysis the resistant genotypes GE796, RAU8, GPU48, and VL324 were grouped along with the HS lines in the cluster D. However in structure analysis results they were grouped with the resistant genotypes. These results showed that structure analysis is more effective and precise in analyzing the finger millet genotypes for blast disease resistance than the phylogenetic analysis. A more detailed marker study would be needed to determine the clear distribution of finger millet genotypes into different groups based on blast resistance. Though similar grouping pattern was observed by both the phylogenetic and structure analysis, the structure bar plot explained the grouping pattern better than the dendrogram by depicting the estimated membership of each variety in each of the populations (K = 1 to 10) and the admixtures could easily be identified.

### Association mapping of blast resistant genes in finger millet genotypes using genic and genomic SSRs

**Figure 4:**
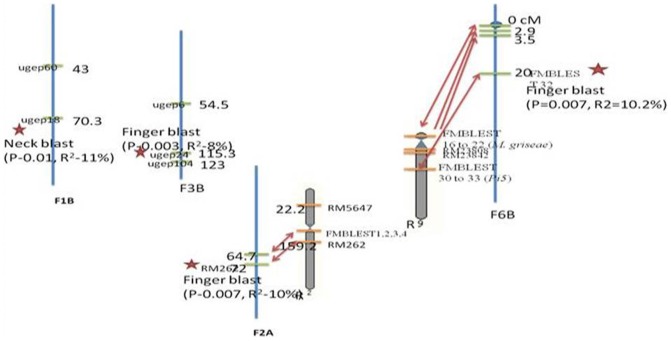
The chromosomal location of the QTLs linked to the blast resistance in finger millet using genic and genomic SSRs (the numerical in cM- centi morgan distance; P- probability of the marker; R^2^- Phenotypic variance explained by the marker; 1B- chromosome 1B, 3B- chromosome 3B; 2A- chromosome 2A).

Association mapping analysis was conducted using the phenotypic data of leaf blast, neck blast, finger blast and the genotypic data of 104 microsatellite markers. Association of SSR marker data with the leaf blast, neck blast and finger blast data resulted in identification of five significant QTLs for finger blast and neck blast at a significant threshold (P) level of ≤0.01 and ≤0.001 by using GLM approach (Table 5). However we did not find any QTLs for leaf blast associated with any of the genic and genomic SSR markers. However, by using MLM approach, seven significant QTLs were identified at significant threshold (P) level of ≤0.01 and ≤0.001 (Table 6). The QTLs for finger blast were strongly associated with the genic SSR primer FMBLEST32 and rice SSR RM262 at a P value of 0.007. The genic SSR marker FMBLEST32 was designed from the *Pi5* rice blast gene which is known for broad spectrum resistance reaction to *M. griseae* [47]. The *Pi5* gene was a dominant locus, which segregated with complete resistance to at least six races belonging to four lineages in the Philippines [47]. Their results showed that *Pi5* gene displayed resistance to diverse isolates and found to have broad spectrum resistance. Hence, same races might be involved in causing the finger blast disease of finger millet. Hence the *Pi5* gene can be a potent resistant gene which confers resistance to the finger blast. The *Pi5* gene encodes proteins carrying three motifs of R genes *viz.,* N-terminal coiled coil (CC) motif, a nucleotide binding (NB) domain, and a leucine-rich repeat (LRR) motif. This showed that there was lot of synteny among the genomes of finger millet and rice. The genomic location of FMBLEST32 marker was found to be on 9^th^ chromosome of rice, while it was on 6B chromosome of finger millet at a distance of 20 cM. The marker FMBLEST32 was linked at a P value of 0.007 and explained phenotypic variance (R^2^) of 10%. In the present study, primers synthesized from *Piz* (FMBLEST26, 27, RM5963, RM3431), *Pi25* (FMBLEST38, 39), *M. griseae* genes (FMBLEST16–22) were also located on the 6B chromosome of finger millet (Figure 4). The studies on fine mapping of blast genes in future may help in finding some of these genes linked to finger blast disease resistance. These results showed that 6B chromosome of finger millet might be hot spot for the genes conferring resistance to the blast. Similar results have also been reported in case of rice where most of the *Pi* genes were located on the 6, 11 and 12 chromosomes [28]. This high level of synteny among the blast genes of finger millet and rice was well supported by the earlier studies, where they found high synteny for the similar genes among the closely related species. So, orthologous genes might be playing important role in finger millet blast resistance. The FMBLEST32 marker generated three alleles at a size of 650, 700 and 710 bp. It was found that most of the resistant genotypes had the 710 bp allele, whereas susceptible genotypes had the 700 bp allele. Hence, the 710 bp allele may be playing important role in the finger blast disease resistance of finger millet.

**Table 5:** The details of the SSR markers linked to blast disease resistance of finger millet genotypes by GLM approach of association mapping (cM- centi morgan).

Blast disease	SSR marker	Probability of marker (Marker p)	Phenotypic variance (R^2^) (%)	Chromosome	Distance	Gene
Finger blast	RM262	0.007	10	2A	72 cM	*Pi-d(t)* blast gene of rice
Finger blast	FMBLEST32	0.007	8	6B	20 cM	*Pi5* blast gene of rice
Neck blast	UGEP18	0.01	11.0	1B	70 cM	*-*
Finger blast	UGEP24	0.003	8.0	3B	115.3 cM	*-*
Finger blast	UGEP81	0.009	7.5	6B	-	*-*

**Table 6:** The details of the SSR markers linked to blast disease resistance of finger millet genotypes by MLM approach of association mapping (cM- centi morgan).

Blast disease	SSR marker	Probability of marker (Marker p)	Phenotypic variance (R^2^) (%)	Chromosome	Distance	Gene
Finger blast	RM262	0.01	5	2A	72 cM	*Pi-d(t)* blast gene of rice
Finger blast	FMBLEST32	0.01	4.5	6B	20 cM	*Pi5* blast gene of rice
Finger blast	UGEP53	0.008	10.5	-	-	*-*
Neck blast	UGEP18	0.009	13	1B	70 cM	*-*
Leaf blast	FMBLEST35	0.009	10	4B	7 cM	*Pi21*
Leaf blast	RM23842	0.009	11	6B	3.5 cM	*M. grisea*
Leaf blast	FMBLEST15	0.006	8	4B	6 cM	NBS-LRR

The marker RM262 linked to finger blast disease of finger millet at a P value of 0.007 and explained phenotypic variance of 8%. The SSR marker RM262 was found to be tightly linked to *Pi-d(t)* blast gene which confers resistance to rice blast. This was located on the long arm of chromosome 2 of rice, while it was located on chromosome 2A of finger millet at a distance of 72 cM. This marker was also close to the genic SSR loci (FMBLEST1, 2, 3, and 4) designed from the finger millet NBS-LRR region, which was 7 cM distance away from them (Figure 4). On the 2^nd^ chromosome of rice, six blast resistant genes *Pi-b* [48], *Pi−14(t)*, *Pi-16(t)*, *Pi-d(t)*, Pi-tq5 and *Pi-25(t)*, have been mapped. The gene *Pi-d(t)* was linked with SSR marker RM262 and RFLP marker G1314 which located on chromosome 2 near the centromere [49], [50]. These results showed that the marker RM262 linked to the finger blast in our study was also mapped to the *Pi-d (t)* gene of rice. Orthologous genes similar to *Pi-d(t)* gene might be playing important role in conferring resistance to the finger blast disease in finger millet. The *Pi-d(t)* gene also close to the *Pi14* and *Pi16* genes, which need to further investigate the role of other genes in the finger millet blast resistance through fine mapping approaches. Thus, the above results showed that in the present scenario where the genome sequence of finger millet is not available, comparative genomics could play a very key role in identification of genes responsible for agriculturally important traits like blast for the finger millet crop improvement. The RM 262 marker produced three alleles at a size of 450, 440 and 600 bp. The allele 440 bp was present in most of the resistant genotypes, whereas 450 bp allele was present in most of the susceptible genotypes. The QTLs for finger blast was strongly associated with two genomic SSRs *i.e.*, UGEP24 and UGEP81. The SSR marker UGEP24 was linked to finger blast at a P value of 0.003 and explained the phenotypic variance of 8%, where as UGEP81 was linked to finger blast at a P value of 0.009 and explained 7.5% of phenotypic variance (Table 5). The marker UGEP24 was located on finger millet chromosome 3B at a distance of 115.3 cM, while the marker UGEP81 was located on 6B chromosome of finger millet. The QTL for neck blast was associated with the genomic SSR marker UGEP18 which was linked at a P value of 0.01 and explained 11% of phenotypic variance. The UGEP18 marker was located on chromosome 1B of finger millet genome at a distance of 70 cM. The chromosomal location of the QTLs linked to the blast resistance was shown in figure 3.

The association mapping was also done using MLM approach of structure software which resulted in identification of seven QTLs (three for finger blast, three for leaf blast and one for neck blast) (Table 6). The QTL for neck blast was linked to a marker UGEP18, common in both GLM and MLM approaches. Likewise the markers FMBLEST32 and RM262 were found associated to finger blast by both the approaches. However, in MLM approach three extra significant QTLs have been detected for leaf blast, which were not detected through GLM approach. The SSR marker UGEP53 was associated to finger blast at a P value of 0.008 by explaining the 10.5% of phenotypic variance. The QTLs for leaf blast was associated with three markers FMBLEST35 (P-0.009, R^2^-10%), RM23842 (P-0.009, R^2^-11%) and FMBLEST15 (P-0.006, R^2^-8%). The functional SSR markers FMBLEST35 and FMBLEST15 were close to each other and located on chromosome 4B of finger millet. The FMBLEST35 marker was developed from *Pi21* gene and FMBLEST15 was from NBS-LRR region. Hence, the coding domains or motifs containing NBS-LRR region like *Pi21* genes might be playing important role in the leaf blast disease resistance. The marker UGEP18 associated to neck blast was found to be linked at P value of 0.009 and explained 13% of phenotypic variance. Thus, the markers found to be associated in both the approaches can be considered as highly effective which and can be used in the marker assisted breeding approaches for introgression of resistant alleles into locally well adapted germplasm.

### The distribution of selected resistant allele amongst global collection of finger millet genotypes

The distribution of resistant allele (710 bp) of FMBLEST32 SSR marker among the finger millet genotypes showed that the resistant allele was present mostly in exotic genotypes. However, among the Indian genotypes, the resistant allele was present in few genotypes, which were from southern and northern part of India. However, no genotype was found to have the resistant allele from the NW Himalayan region of India. The SSR marker RM262 comprised of two alleles, of which the allele 440 bp was present in most of the resistant genotypes such as exotic genotypes. In India, NW Himalayan genotypes had this resistant allele when compared to the genotypes from other parts of India. The resistant alleles found in the present study can be further used for cloning of the full length gene, fine mapping and their further use in the marker assisted breeding programmes for introgression of blast resistant alleles into locally well adapted cultivars.

## Conclusions

The present study is the first report in development of functional SSR markers for finger millet blast resistance genes using comparative genomic analysis with rice for genetic diversity, population structure and association mapping studies. The present investigation resulted in development of 58 functional SSR markers for several important blast resistant genes. Association mapping analysis with 104 SSRs resulted in identification of five QTLs for blast resistance (four for finger blast and one for neck blast) by GLM approach. However, seven markers were associated to the leaf, neck and finger blast by MLM approach. The three markers RM262, FMBLEST32 and UGEP18 were found to be linked to blast disease by both GLM and MLM approaches. The results obtained from association mapping showed that 2^nd^ and 6^th^ chromosomes of finger millet might be the major hub of finger blast and neck blast resistant genes. The identified markers can be further used in fine mapping, cloning of full length blast genes and marker assisted breeding programmes of finger millet.

## Supporting Information

Figure S1 **The hypothetical positions of the blast primers on the finger millet and rice chromosomes (R- Rice, F- Finger millet, Number indicates the distance in centi morgan).**
(DOC)

Table S1 **The list of finger millet genotypes under the study along with their origin and source of collection.**
(DOC)

Table S2 **The details of the blast genic SSR loci along with their contig number, repeat motif, product size and gene homology.**
(DOC)
