# Supplementary material for: Comparative Genomics and Association Mapping Approaches for Blast Resistant Genes in Finger Millet Using SSRs
Source: PLoS One. 2014 Jun 10;9(6):e99182. doi: 10.1371/journal.pone.0099182 (PMC4051690; doi:10.1371/journal.pone.0099182)
Supplement: Figure S1 — The hypothetical positions of the blast primers on the finger millet and rice chromosomes (R- Rice, F- Finger millet, Number indicates the distance in centi morgan). (DOC) [file pone.0099182.s001.doc]

**Supplemental figure S1**

The hypothetical positions of the blast primers on the finger millet and rice chromosomes (R- Rice, F- Finger millet, Number indicates the distance in centi morgan)

**
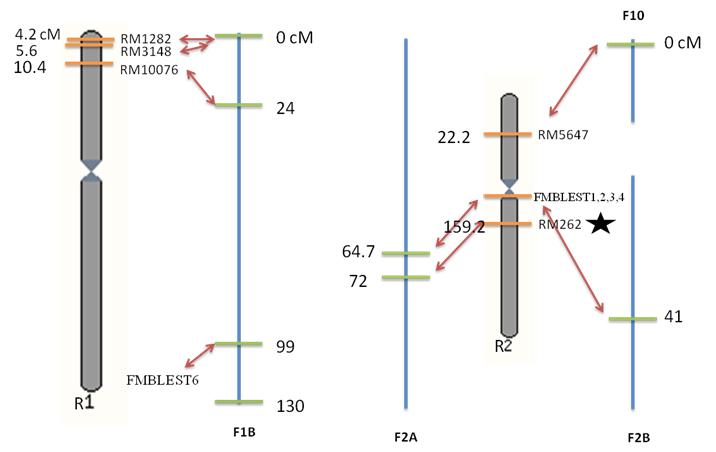
**

**
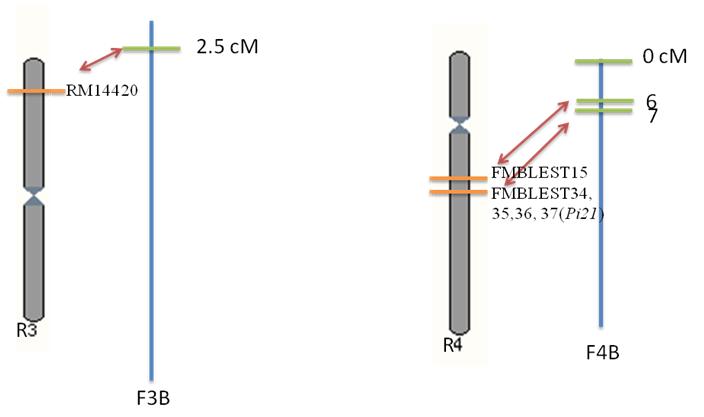
**

**
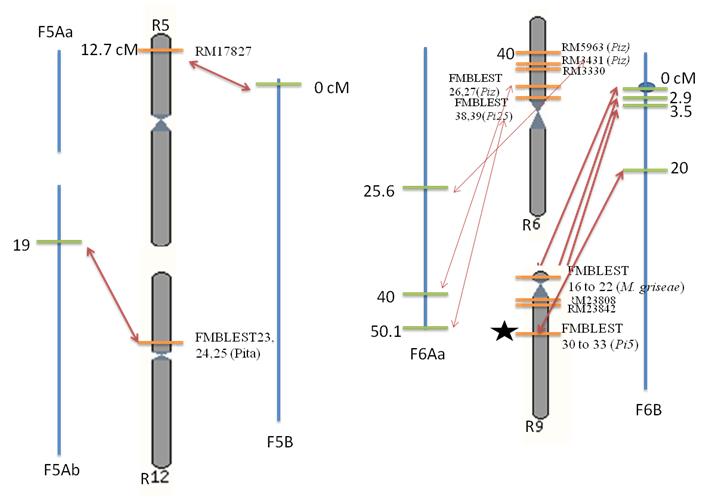
**

**
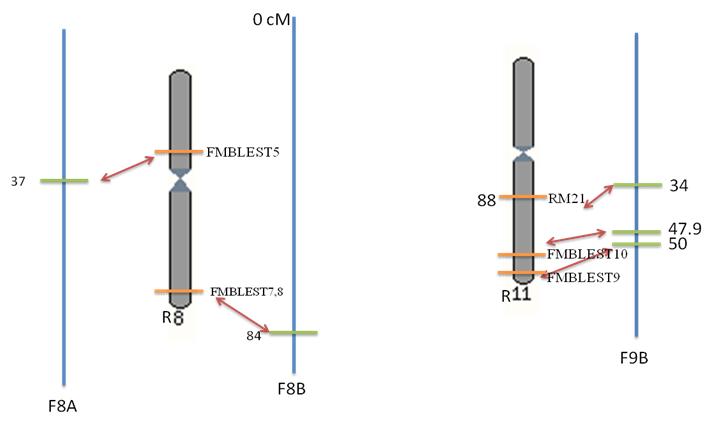
**
