# Supplementary material for: Comparative Genomics and Association Mapping Approaches for Blast Resistant Genes in Finger Millet Using SSRs
Source: PLoS One. 2014 Jun 10;9(6):e99182. doi: 10.1371/journal.pone.0099182 (PMC4051690; doi:10.1371/journal.pone.0099182)
Supplement: Table S1 — The list of finger millet genotypes under the study along with their origin and source of collection. (DOC) [file pone.0099182.s002.doc]

**Supplemental table S1**

The list of finger millet genotypes under the study along with their origin and source of collection

| S. No. | Genotype | Origin | Source |
| --- | --- | --- | --- |
| 1 | IE3391 | Zimbabwe | ICRISAT mini-core |
| 2 | IE4491 | Zimbabwe | ICRISAT mini-core |
| 3 | IE7320 | Kenya | ICRISAT mini-core |
| 4 | IE4797 | Maldives | ICRISAT mini-core |
| 5 | IE3077 | India | ICRISAT mini-core |
| 6 | IE4121 | Uganda | ICRISAT mini-core |
| 7 | IE4073 | Uganda | ICRISAT mini-core |
| 8 | IE2710 | Malawi | ICRISAT mini-core |
| 9 | IE2872 | Zambia | ICRISAT mini-core |
| 10 | IE5066 | Senegal | ICRISAT mini-core |
| 11 | IE4570 | Zimbabwe | ICRISAT mini-core |
| 12 | VR708 | India | VPKAS, Almora, India |
| 13 | IE5537 | Nepal | ICRISAT mini-core |
| 14 | IE2043 | ICRISAT | ICRISAT mini-core |
| 15 | IE3317 | Zimbabwe | ICRISAT mini-core |
| 16 | IE2034 | India | ICRISAT mini-core |
| 17 | IE2790 | Malawi | ICRISAT mini-core |
| 18 | IE2871 | Zambia | ICRISAT mini-core |
| 19 | IE5306 | Zimbabwe | ICRISAT mini-core |
| 20 | IE4816 | India | ICRISAT mini-core |
| 21 | IE4497 | Zimbabwe | ICRISAT mini-core |
| 22 | IE6240 | Zimbabwe | ICRISAT mini-core |
| 23 | IE6421 | Uganda | ICRISAT mini-core |
| 24 | IE6165 | Nepal | ICRISAT mini-core |
| 25 | IE4028 | Uganda | ICRISAT mini-core |
| 26 | IE6221 | Nepal | ICRISAT mini-core |
| 27 | IE6337 | Zimbabwe | ICRISAT mini-core |
| 28 | IE6537 | Nigeria | ICRISAT mini-core |
| 29 | IE6059 | Nepal | ICRISAT mini-core |
| 30 | IE2821 | Nepal | ICRISAT mini-core |
| 31 | IE4673 | ICRISAT | ICRISAT mini-core |
| 32 | IE5367 | Kenya | ICRISAT mini-core |
| 33 | IE3973 | Uganda | ICRISAT mini-core |
| 34 | IE3104 | India | ICRISAT mini-core |
| 35 | IE2572 | Kenya | ICRISAT mini-core |
| 36 | IE2911 | Zambia | ICRISAT mini-core |
| 37 | IE6350 | Zimbabwe | ICRISAT mini-core |
| 38 | IE2430 | Kenya | ICRISAT mini-core |
| 39 | IE5817 | Nepal | ICRISAT mini-core |
| 40 | IE4734 | India | ICRISAT mini-core |
| 41 | IE2042 | India | ICRISAT mini-core |
| 42 | IE2217 | India | ICRISAT mini-core |
| 43 | IE5091 | Zimbabwe | ICRISAT mini-core |
| 44 | IE2619 | Malawi | ICRISAT mini-core |
| 45 | IE2457 | Kenya | ICRISAT mini-core |
| 46 | IE501 | India | ICRISAT mini-core |
| 47 | IE2437 | Kenya | ICRISAT mini-core |
| 48 | IE4622 | Zimbabwe | ICRISAT mini-core |
| 49 | PRM1 | India | ICRISAT mini-core |
| 50 | IE3475 | India | ICRISAT mini-core |
| 51 | IE4795 | Zimbabwe | ICRISAT mini-core |
| 52 | IE4565 | Zimbabwe | ICRISAT mini-core |
| 53 | IE4757 | India | ICRISAT mini-core |
| 54 | IE6154 | Nepal | ICRISAT mini-core |
| 55 | IE3618 | NA | ICRISAT mini-core |
| 56 | IE4646 | Zimbabwe | ICRISAT mini-core |
| 57 | IE4671 | India | ICRISAT mini-core |
| 58 | IE3470 | India | ICRISAT mini-core |
| 59 | IE5106 | Zimbabwe | ICRISAT mini-core |
| 60 | IE4057 | Uganda | ICRISAT mini-core |
| 61 | IE7079 | Kenya | ICRISAT mini-core |
| 62 | IE2957 | Germany | ICRISAT mini-core |
| 63 | IE3945 | Uganda | ICRISAT mini-core |
| 64 | IE5870 | Nepal | ICRISAT mini-core |
| 65 | IE4545 | Zimbabwe | ICRISAT mini-core |
| 66 | IE2312 | India | ICRISAT mini-core |
| 67 | IE6294 | Zimbabwe | ICRISAT mini-core |
| 68 | IE3392 | Zimbabwe | ICRISAT mini-core |
| 69 | IE6473 | Uganda | ICRISAT mini-core |
| 70 | IE6326 | Zimbabwe | ICRISAT mini-core |
| 71 | IE7018 | Kenya | ICRISAT mini-core |
| 72 | IE3045 | India | ICRISAT mini-core |
| 73 | IE2296 | India | ICRISAT mini-core |
| 74 | IE5201 | India | ICRISAT mini-core |
| 75 | IE6082 | Nepal | ICRISAT mini-core |
| 76 | IE3696 | ICRISAT | ICRISAT mini-core |
| 77 | VHC3997 | India | VPKAS, Almora, India |
| 78 | IE3698 | Uganda | ICRISAT core |
| 79 | IE3699 | Uganda | ICRISAT core |
| 80 | IE3700 | ICRISAT | ICRISAT core |
| 81 | IE3701 | ICRISAT | ICRISAT core |
| 82 | IE3702 | ICRISAT | ICRISAT core |
| 83 | IE3703 | ICRISAT | ICRISAT core |
| 84 | IE3770 | ICRISAT | ICRISAT core |
| 85 | IE3769 | ICRISAT | ICRISAT core |
| 86 | IE3704 | ICRISAT | ICRISAT core |
| 87 | IE3797 | ICRISAT | ICRISAT core |
| 88 | IE3768 | ICRISAT | ICRISAT core |
| 89 | IE3791 | Uganda | ICRISAT core |
| 90 | IE3793 | ICRISAT | ICRISAT core |
| 91 | IE3775 | ICRISAT | ICRISAT core |
| 92 | IE3794 | ICRISAT | ICRISAT core |
| 93 | IE3772 | ICRISAT | ICRISAT core |
| 94 | IE3771 | ICRISAT | ICRISAT core |
| 95 | IE3795 | Uganda | ICRISAT core |
| 96 | IE6514 | ICRISAT | ICRISAT core |
| 97 | IE3712 | ICRISAT | ICRISAT core |
| 98 | GE724 | UP, India | GBPUA & T, Pantnagar |
| 99 | GE1621 | KN, India | GBPUA & T, Pantnagar |
| 100 | GE1298 | UP, India | GBPUA & T, Pantnagar |
| 101 | GE1583 | UP, India | GBPUA & T, Pantnagar |
| 102 | GE2447 | UP, India | GBPUA & T, Pantnagar |
| 103 | GE4692 | Uganda | GBPUA & T, Pantnagar |
| 104 | GE4811 | Malawi | GBPUA & T, Pantnagar |
| 105 | GE1899 | UP, India | GBPUA & T, Pantnagar |
| 106 | GE1680 | UP, India | GBPUA & T, Pantnagar |
| 107 | GE763 | UP, India | GBPUA & T, Pantnagar |
| 108 | GE4440 | Orissa, India | GBPUA & T, Pantnagar |
| 109 | GE1240 | TamilNadu, India | GBPUA & T, Pantnagar |
| 110 | GE2238 | TamilNadu, India | GBPUA & T, Pantnagar |
| 111 | GE1235 | UP, India | GBPUA & T, Pantnagar |
| 112 | GE1936 | UP, India | GBPUA & T, Pantnagar |
| 113 | GE1093 | UP, India | GBPUA & T, Pantnagar |
| 114 | GE1146 | UP, India | GBPUA & T, Pantnagar |
| 115 | GE2063 | TamilNadu, India | GBPUA & T, Pantnagar |
| 116 | GE116 | Madhya Pradesh, India | GBPUA & T, Pantnagar |
| 117 | GE2154 | UP, India | GBPUA & T, Pantnagar |
| 118 | GE128 | Bihar, India | GBPUA & T, Pantnagar |
| 119 | GE356 | UP, India | GBPUA & T, Pantnagar |
| 120 | GE2136 | TamilNadu, India | GBPUA & T, Pantnagar |
| 121 | GE384 | AP, India | GBPUA & T, Pantnagar |
| 122 | GE390 | TamilNadu, India | GBPUA & T, Pantnagar |
| 123 | GE3147 | KN, India | GBPUA & T, Pantnagar |
| 124 | GE1537 | UP, India | GBPUA & T, Pantnagar |
| 125 | GE619 | TamilNadu, India | GBPUA & T, Pantnagar |
| 126 | GE514 | UP, India | GBPUA & T, Pantnagar |
| 127 | GE4449 | Orissa, India | GBPUA & T, Pantnagar |
| 128 | GE1437 | UP, India | GBPUA & T, Pantnagar |
| 129 | GE2624 | UP, India | GBPUA & T, Pantnagar |
| 130 | GE2471 | UP, India | GBPUA & T, Pantnagar |
| 131 | GE4404 | Bihar, India | GBPUA & T, Pantnagar |
| 132 | VHC3944 | India | VPKAS, Almora, India |
| 133 | VHC3870 | India | VPKAS, Almora, India |
| 134 | VHC3962 | India | VPKAS, Almora, India |
| 135 | VHC3996 | India | VPKAS, Almora, India |
| 136 | VHC3991 | India | VPKAS, Almora, India |
| 137 | VHC3984 | India | VPKAS, Almora, India |
| 138 | VHC3980 | India | VPKAS, Almora, India |
| 139 | VHC3970 | India | VPKAS, Almora, India |
| 140 | VHC3951 | India | VPKAS, Almora, India |
| 141 | VHC3907 | India | VPKAS, Almora, India |
| 142 | VHC3908 | India | VPKAS, Almora, India |
| 143 | VHC3887 | India | VPKAS, Almora, India |
| 144 | VHC3881 | India | VPKAS, Almora, India |
| 145 | VHC3956 | India | VPKAS, Almora, India |
| 146 | VHC3930 | India | VPKAS, Almora, India |
| 147 | VHC3876 | India | VPKAS, Almora, India |
| 148 | VHC3903 | India | VPKAS, Almora, India |
| 149 | VHC3895 | India | VPKAS, Almora, India |
| 150 | VHC3898 | India | VPKAS, Almora, India |
| 151 | VHC3893 | India | VPKAS, Almora, India |
| 152 | VHC4013 | India | VPKAS, Almora, India |
| 153 | VHC3873 | India | VPKAS, Almora, India |
| 154 | VHC3865 | India | VPKAS, Almora, India |
| 155 | VHC3911 | India | VPKAS, Almora, India |
| 156 | VHC3972 | India | VPKAS, Almora, India |
| 157 | VHC3917 | India | VPKAS, Almora, India |
| 158 | VHC3939 | India | VPKAS, Almora, India |
| 159 | GE496 | MP, India | GBPUA & T, Pantnagar |
| 160 | GE669 | Africa | GBPUA & T, Pantnagar |
| 161 | GE796 | NA | GBPUA & T, Pantnagar |
| 162 | RAU8 | India | GBPUA & T, Pantnagar |
| 163 | GPU28 | NA | GBPUA & T, Pantnagar |
| 164 | GPU48 | NA | GBPUA & T, Pantnagar |
| 165 | KM252 | NA | GBPUA & T, Pantnagar |
| 166 | VR708 | India | VPKAS, Almora, India |
| 167 | VL149 | India | VPKAS, Almora, India |
| 168 | VL324 | India | VPKAS, Almora, India |
| 169 | VL333 | India | VPKAS, Almora, India |
| 170 | VL201 | India | VPKAS, Almora, India |
| 171 | VL204 | India | VPKAS, Almora, India |
| 172 | GE5192 | Uganda | UAS, Bangalore |
| 173 | VL315 | India | VPKAS, Almora, India |
| 174 | GE4440 | Orissa, India | UAS, Bangalore |
| 175 | GPHCPB1 | Pantnagar, India | GBPUA & T, Pantnagar |
| 176 | GPHCPB2 | Pantnagar, India | GBPUA & T, Pantnagar |
| 177 | GPHCPB4 | Pantnagar, India | GBPUA & T, Pantnagar |
| 178 | GPHCPB5 | Pantnagar, India | GBPUA & T, Pantnagar |
| 179 | GPHCPB10 | Pantnagar, India | GBPUA & T, Pantnagar |
| 180 | GPHCPB11 | Pantnagar, India | GBPUA & T, Pantnagar |
| 181 | GPHCPB13 | Pantnagar, India | GBPUA & T, Pantnagar |
| 182 | GPHCPB20 | Pantnagar, India | GBPUA & T, Pantnagar |
| 183 | GPHCPB25 | Pantnagar, India | GBPUA & T, Pantnagar |
| 184 | GPHCPB26 | Pantnagar, India | GBPUA & T, Pantnagar |
| 185 | GPHCPB27 | Pantnagar, India | GBPUA & T, Pantnagar |
| 186 | GPHCPB29 | Pantnagar, India | GBPUA & T, Pantnagar |
| 187 | GPHCPB30 | Pantnagar, India | GBPUA & T, Pantnagar |
| 188 | IE2589 | America | ICRISAT mini-core |
| 189 | IE3614 | NA | ICRISAT mini-core |
| 190 | VHC3697 | India | VPKAS, Almora, India |

NA- Not available
