# Supplementary material for: Comparative Genomics and Association Mapping Approaches for Blast Resistant Genes in Finger Millet Using SSRs
Source: PLoS One. 2014 Jun 10;9(6):e99182. doi: 10.1371/journal.pone.0099182 (PMC4051690; doi:10.1371/journal.pone.0099182)
Supplement: Table S2 — The details of the blast genic SSR loci along with their contig number, repeat motif, product size and gene homology. (DOC) [file pone.0099182.s003.doc]

**Supplemental table S2**

The details of the blast genic SSR loci along with their contig number, repeat motif, product size and gene homology.

| Primer | Contig number | Repeat motif | Expected product size | Gene homology | Sequence of primer |
| --- | --- | --- | --- | --- | --- |
| FMBLESTSSR1 | EU075263.1 | (GGTGC)2 | 163 | Finger millet NBS-LRR region | F 5’ GGGAGCTTGTTGGAGAAGATAA 3’ |
|  |  |  |  |  | R 5’ TCTTTGAGCAGATTGTCCCATA 3’ |
| FMBLESTSSR2 | EU075263.1 | (TGGCG)2 | 398 | Finger millet NBS- LRR region | 5’ TATGGGACAATCTGCTCAAAGA 3’ |
|  |  |  |  |  | 5’ AGAAGGCAGGTGAACGTAACTC 3’ |
| FMBLESTSSR3 | EU075262.1 | (TGGCG)2 | 384 | Finger millet NBS- LRR region | 5’ GGATGAAGTGAGATTCCCCATA 3’ |
|  |  |  |  |  | 5’ CAAGCTCTTCTGTGTTGCACTC 3’ |
| FMBLESTSSR4 | EU075259.1 | (TGGCG)2 | 397 | Finger millet NBS- LRR region | 5’ TTGTCTGGGATGACTTGAACAG 3’ |
|  |  |  |  |  | 5’ AGCTCGTGGTACTTATGGAAGG 3’ |
| FMBLESTSSR5 | DQ272576.1 | (TAA)4 | 342 | Rice NBS-LRR region | 5’ AGTGTATCAAGAGCAAGGGGAA 3’ |
|  |  |  |  |  | 5’ CCACATTTACGAACAATCTCCA 3’ |
| FMBLESTSSR6 | AK106251.1 | (GCTCA)2 | 131 | Rice NBS – LRR region | 5’ GTAGGAAAAGATGGAGCAGTGG 3’ |
|  |  |  |  |  | 5’ AACTTGAGCTTGGATTGATGGT 3’ |
| FMBLESTSSR7 | AK099583.1 | (TGAAA)2 | 348 | Rice NBS- LRR region | 5’ ATAATGGAAACCCTTCAACCCT 3’ |
|  |  |  |  |  | 5’ CTTGCAGAAAATTGCACAGAAG 3’ |
| FMBLESTSSR8 | AK099583.1 | (TATGGT)2 | 364 | Rice NBS- LRR region | 5’ AGAAGCCAATCTCCATCAGAAA 3’ |
|  |  |  |  |  | 5’ AAATAACTGCACTGGGGTGTTC 3’ |
| FMBLESTSSR9 | AF220745.1 | (TTGAT)2 | 246 | Rice NBS- LRR region | 5’ ACCTTCAAAGCAAACTCAAAGC 3’ |
|  |  |  |  |  | 5’ TGTTCATGCTCCTCCAAAGTAA 3’ |
| FMBLESTSSR10 | EU075225.1 | (TGGAG)2 | 334 | Finger millet NBS- LRR region | 5’ AAGGGAGCATTTAGCAACAAAG 3’ |
|  |  |  |  |  | 5’ CATCATCGGACATCAATTCAAC 3’ |
| FMBLESTSSR11 | U76215.1 | (AGGGAG)2 | 179 | NBS- LRR region | 5’ CATTGAAACCATGTTGATGTCC 3’ |
|  |  |  |  |  | 5’ CTCATTCTCTTCCTCCTTGCTC 3’ |
| FMBLESTSSR12 | U76215.1 | (TGAGA)2 | 398 | NBS-LRR region | 5’ TACACTTCACCAGAGACGAGGA 3’ |
|  |  |  |  |  | 5’ TGTCATTTATCTCCGACCACAA 3’ |
| FMBLESTSSR13 | U76215.1 | (CTATTG)2 | 380 | NBS- LRR region | 5’ GCTAAAGACAAAGAGTGCCCAT 3’ |
|  |  |  |  |  | 5’ AAATATCCCTCTGCCACTTCCT 3’ |
| FMBLESTSSR14 | U76215.1 | (GTTAAT)2 | 300 | NBS- LRR region | 5’ ATTTGTTACGGCAGGTTTGTCT 3’ |
|  |  |  |  |  | 5’ GCGTGTAGGAGAAGCAAGAAGT 3’ |
| FMBLESTSSR15 | GU301915.1 | (GCAAG)2 | 248 | NBS- LRR region | 5’ GATAGTGAGGAAAGTCCGATGG 3’ |
|  |  |  |  |  | 5’ CCCCTTGTCTTCTTTTGCATAC 3’ |
| FMBLESTSSR16 | AB026819.1 | (AATAG)2 | 364 | Rice *M. griseae* genes | 5’ GGGGAACCAGGACTTTTACTTT 3’ |
|  |  |  |  |  | 5’ TTGTACTTTCGTGTAAGGTGCC 3’ |
| FMBLESTSSR17 | AB026819.1 | (CGAGT)2 | 281 | Rice *M. griseae* genes | 5’ AATAAGCGGAGGAAAAGAAACC 3’ |
|  |  |  |  |  | 5’ TGGCCGGTATTTAGCTTTAGAA 3’ |
| FMBLESTSSR18 | AB026819.1 | (GCAAC)2 | 201 | Rice *M. griseae* genes | 5’ AGCAGTTGGACGTGGGTTAG 3’ |
|  |  |  |  |  | 5’ GGTGGTGGATCGTTAAGAAGAA 3’ |
| FMBLESTSSR19 | AB026819.1 | (TTGATT)2 | 266 | Rice *M. griseae* genes | 5’ CCAACTTCTGGTTTTAACGTCC 3’ |
|  |  |  |  |  | 5’ GTGGTAACTTTTCTGGCACCTC 3’ |
| FMBLESTSSR20 | AB026819.1 | (AGTAG)2 | 369 | Rice *M. griseae* genes | 5’ AGAGGAACCGCTCATTCAGATA 3’ |
|  |  |  |  |  | 5’ GGGGTAGAAAGTCTGGGAAATC 3’ |
| FMBLESTSSR21 | AB026819.1 | (T)11 | 330 | Rice *M. griseae* genes | 5’ AAGGGGTGCAGGGTAAGTAAGT 3’ |
|  |  |  |  |  | 5’ TATAAATAGCGTCAACGTCCCG 3’ |
| FMBLESTSSR22 | AB026819.1 | (CGCGG)3 | 387 | Rice *M. griseae* genes | 5’ CGGGACGTTGACGCTATTTAT 3’ |
|  |  |  |  |  | 5’GCTGTCAAATCAGCAACTAACG3’ |
| FMBLESTSSR23 | HE580106.1 | (GCC)4 | 274 | Pi-ta | 5’AGTCATCTAGCAGGTGTCGGAG3’ |
|  |  |  |  |  | 5’AGCTAACGACCCAGCTCCTC3’ |
| FMBLESTSSR24 | HE580106.1 | (GCG)4 | 354 | Pi-ta | 5’TTTGCATCGAAATATCCCTACC3’ |
|  |  |  |  |  | 5’CTCTAAGGAGACAGGGTTGGAA3’ |
| FMBLESTSSR25 | HE580106.1 | (TGA)4 | 323 | Pi-ta | 5’CAGGTAAAGATAGAAGCCACGG3’ |
|  |  |  |  |  | 5’CCCAGTTCTCTGGATCAACTCT3’ |
| FMBLESTSSR26 | HE580189.1 | (ATTGGA)2 | 278 | Piz | 5’ATCTGCAAGAAATCAAGGTGGT3’ |
|  |  |  |  |  | 5’TAGAAATAGGCGAGTGCAGGTT3’ |
| FMBLESTSSR27 | HE580189.1 | (AAAGT)2 | 275 | Piz | 5’TGCTTCCACTGCTACATTGACT3’ |
|  |  |  |  |  | 5’CAGACCATACCAAAGATCACGA3’ |
| FMBLESTSSR28 | JQ657221.1 | (AAATGG)2 | 323 | Rice NBS – LRR region | 5’CAGAACGCCTGTATTTTCATCA3’ |
|  |  |  |  |  | 5’TCTGACGTTGATTTTGATGAGC3’ |
| FMBLESTSSR29 | JQ657131.1 | (AATGG)2 | 350 | Rice NBS – LRR region | 5’AGCTTTCGGAGGGAAGTTAGAC3’ |
|  |  |  |  |  | 5’ACGGTTATGAGAAGTGGAAATG3’ |
| FMBLESTSSR30 | EU869185.1 | (CTGGG)2 | 273 | Pi5-1 | 5’AGTTGGGAGGAAATCCAGACTA3’ |
|  |  |  |  |  | 5’TACCACGAACTAAACACAGCCA3’ |
| FMBLESTSSR31 | EU869185.1 | (CT)7 | 121 | Pi5-1 | 5’TTTCTATGCGGAGGAAATGAGT3’ |
|  |  |  |  |  | 5’CCCTCCTCCTCCTCCTCTAGT3’ |
| FMBLESTSSR32 | EU869185.1 | (GCC)4 | 391 | Pi5-1 | 5’TCCTTTGCCACTTTATCCAATC3’ |
|  |  |  |  |  | 5’GAATGGGGTATGAATGGAACAC3’ |
| FMBLESTSSR33 | EU869185.1 | (AAAGCA)2 | 370 | Pi5-1 | 5’TAATGTAAAGGGTCGGAATCGT3’ |
|  |  |  |  |  | 5’CCGGATTAGCAGAGTTATTTGG3’ |
| FMBLESTSSR34 | AB430853.1 | (AT)5 | 398 | Pi-21 | 5’AAGAAACATTGCACTCATGTGG3’ |
|  |  |  |  |  | 5’CATCAGCGTTTGTGGTGTACTT3’ |
| FMBLESTSSR35 | AB430853.1 | (GAAAGC)2 | 399 | Pi-21 | 5’TGTGGTCCTGAAACCATGTTAG3’ |
|  |  |  |  |  | 5’ACGTGAAGAACAACAGGGTGAT3’ |
| FMBLESTSSR36 | AB430853.1 | (AAGCCC)4 | 257 | Pi-21 | 5’CTCGCAGTGACAGGGCTT3’ |
|  |  |  |  |  | 5’GTGTTTGTGTTGTGTGCTGTGT3’ |
| FMBLESTSSR37 | AB430853.1 | (TTTG)4 | 256 | Pi-21 | 5’TCATAAAGAAGGGGAGAAACGA3’ |
|  |  |  |  |  | 5’AAGTCCTTGCTTGGGCTAGAG3’ |
| FMBLESTSSR38 | JQ838019.1 | (GGATGG)2 | 362 | Pi25 | 5’ACGCCCTTCAGATCATACCTAA3’ |
|  |  |  |  |  | 5’AATTACAGAGGCTTGGTCGAAA3’ |
| FMBLESTSSR39 | JQ838019.1 | (TCTGT)2 | 280 | Pi25 | 5’GCGGACAATTCCTTATCTCATT3’ |
|  |  |  |  |  | 5’GCGGACAATTCCTTATCTCATT3’ |
| FMBLESTSSR40 | JQ838019.1 | (GGATGG)2 | 230 | NA | 5’GCTAGGGAGATTTACGACGATGAC3’ |
|  |  |  |  |  | 5’ATGGTGTTGCACTCAGAAGGAC3’ |
| FMBLESTSSR41 | AB430853.1 | (AAGCCC)4 | 320 | NA | 5’GACTAACAGGAGAATGCTCATGG3’ |
|  |  |  |  |  | 5’TGAGTGAAGGTAAGCACTTCCTG3’ |
| FMBLESTSSR42 | AB430853.1 | NA | 110 | NA | 5’CTTTCAGTTGAAGATGTGGCACTG3’ |
|  |  |  |  |  | 5’TGACAATACGTCTGCCAATACCGA3’ |
| FMBLESTSSR43 | EU869185.1 | NA | 420 | NA | 5’GAGGCCCACACACTTCTTAGCGA3’ |
|  |  |  |  |  | 5’GGAGTATGGGTAGTGGAACCAGTG3’ |
| RM262 | Rice SSR loci | (CT)16 | 270 | Pi14/16 | 5’CATTCCGTCTCGGCTCAACT3’ |
|  |  |  |  |  | 5’CAGAGCAAGGTGGCTTGC3’ |
| RM3431 | Rice SSR loci | (CT)18 | 390 | Piz | 5’ATCCAAATCCAATGGTGC3’ |
|  |  |  |  |  | 5’GCGAAAGGGAACATTCTG3’ |
| RM3330 | Rice SSR loci | (CT)15 | 250 | Pi40 | 5’ATTATTCCCCTCTTCCGCTC3’ |
|  |  |  |  |  | 5’AAGAAACCCTCGGATTCCTG3’ |
| RM5647 | Rice SSR loci | (AAG)16 | 500 | Pi36 | 5’ACTCCGACTGCAGTTTTTGC3’ |
|  |  |  |  |  | 5’AACTTGGTCGTGGACAGTGC3’ |
| RM1282 | Rice SSR loci | NA | 340 | NA | 5’AAGCATGACAGCTGCAAGAC3’ |
|  |  |  |  |  | 5’GGGGATGAAGGGTAATTTCG3’ |
| RM5963 | Rice SSR loci | (CAG)9 | 430 | Pi2, PiZ | 5’CGAAAAGTGGGAAGCAAATG3’ |
|  |  |  |  |  | 5’GCGTACCCCTAGTGGCTGTA3’ |
| RM3148 | Rice SSR loci | (CA)20 | 155 | NA | 5’GACTATTGCTCGAACACTTTG3’ |
|  |  |  |  |  | 5’TTGTCTGCTTTGGTATTTGC3’ |
| RM10076 | Rice SSR loci | (CT)27 | 187 | NA | 5’CTAGCAGCTGTCTGCGACACACG3’ |
|  |  |  |  |  | 5’CCGAGGTGTTATGCCAATCTCTATGG3’ |
| RM14420 | Rice SSR loci | (CT)17 | 198 | NA | 5’AACGCTCTCTCAATCTCTCTGC3’ |
|  |  |  |  |  | 5’GTCAATGATGGTCACTTGATGC3’ |
| RM17827 | Rice SSR loci | (AT)12 | 312 | NA | 5’CGGCCCAAGAAGGATATGAAGG3’ |
|  |  |  |  |  | 5’GTCAAACTTCACCGCTCGTTGG3’ |
| RM23842 | Rice SSR loci | (AGAT)6 | 262 | NA | 5’TATAGGGTGCTACGTGACGATACGG3’ |
|  |  |  |  |  | 5’GCGCAAATCCAGTCAAGTCTCC3’ |
| RM20827 | Rice SSR loci | (AG)11 | 93 | NA | 5’GTAGTAGCATGTCCATTGTTGAGG3’ |
|  |  |  |  |  | 5’GCATTCCTACTTCAGGTTTCTGC3’ |
| RM23808 | Rice SSR loci | (AGAT)6 | 452 | NA | 5’AAGTGCTACGTGGCGATTTAGG3’ |
|  |  |  |  |  | 5’TTGAGTTATTTGCGAGGCTACG3’ |
| RM23823 | Rice SSR loci | (TA)27 | 148 | NA | 5’AACTTGGACGTGACACTAACAAGAGC3’ |
|  |  |  |  |  | 5’GCGATACGGGAGCGTGTATAGG3’ |

NA- Not available
